# “I Have Guys Call Me and Say ‘I Can’t Be the Victim of Domestic Abuse’”: Exploring the Experiences of Telephone Support Providers for Male Victims of Domestic Violence and Abuse

**DOI:** 10.1177/0886260520944551

**Published:** 2020-07-29

**Authors:** Benjamin Hine, Elizabeth A. Bates, Sarah Wallace

**Affiliations:** 1 University of West London, Brentford, UK; 2 University of Cumbria, Carlisle, UK; 3 University of South Wales, Pontypridd, UK

**Keywords:** domestic violence, intervention/treatment, disclosure of domestic violence, perceptions of domestic violence

## Abstract

While previous studies have begun to provide evidence on the experiences of male victims of domestic violence and abuse (DVA), current understanding in this area is still limited, and subject to narrow methods of inquiry. Moreover, little is known regarding the challenges of providing support to men in abusive relationships, and how barriers to effective service engagement are experienced by both men and service practitioners. This is an important area for exploration, as the gender-specific experiences and needs of men have been historically overlooked within academic research and service provision. The present study therefore had two principal aims: first, to provide more detailed information regarding the nature and context of abuse toward, and help-seeking experiences of, male victims, and second, to explore the experiences of those supporting abused men. Semi-structured interviews were conducted with four call handlers at a U.K. domestic abuse charity supporting male victims. Transcribed interviews were subjected to thematic analysis, revealing a superordinate theme of *stereotypes and expectations of men* which affected all the other three overarching and eight subthemes, including those detailing the range and severity of abuse suffered, the role of family and friends, barriers to reporting for abused men, and challenges in supporting them. Implications for services working with male victims of DVA are discussed: centered around the need for recognition, increased awareness, increased resourcing, and the provision of gender-inclusive services catering for the gender-specific needs of men.

## Introduction

Much of the early research exploring domestic violence and abuse (DVA) posited that such violence results from men’s desire to control and dominate, rooted in historically and socially constructed patriarchal values which emphasize and facilitate male privilege. This valuable body of work has been instrumental in highlighting and exploring the experiences of abused women, and their support needs. However, the pervasiveness of this approach has led to the identification of a “gendered paradigm” (Dutton & Nicholls, 2005; Dutton & White, 2013) or “domestic violence stereotype” (E. A. Bates et al., 2019; Hine, 2019) within the literature which uniformly describes DVA as physical abuse perpetrated by men toward women, and which excuses female violence as enacted primarily in self-defense (Dutton & Corvo, 2006).

As a result, male victims have often been overlooked, and remained a “hidden” victim group, despite some researchers, and government statistics, evidencing their existence for decades (Cook, 2009). Indeed, more recent research, which positions DVA in the context of other family violence, has provided convincing evidence of female aggression (Archer, 2000; E. A. Bates et al., 2014; Hines & Douglas, 2009) and control (E. A. Bates & Graham-Kevan, 2016; Carney & Barner, 2012), and “intimate partner terrorism” (Hines & Douglas, 2010a, 2010b), and the prevalence of bidirectional DVA (Langhinrichsen-Rohling et al., 2012). Moreover, studies are beginning to highlighting the prevalence of DVA within same-sex relationships, including against GBT (gay, bisexual, and transgender) men (Bacchus et al., 2017). As such, literature examining the experiences, outcomes, and help-seeking behavior of male victims, while still sparse (Morgan et al., 2014), is beginning to develop into a significant body of research.

Studies exploring the prevalence of female-on-male DVA have identified substantial physical aggression toward men, including kicking, biting, choking, scratching, and the use of weapons (Drijber et al., 2013; Hines et al., 2007). Importantly, studies have shown that around one third of abused men sustain *serious* injury as a result of female perpetrated abuse (Hines & Douglas, 2010a). Qualitative studies offer further in-depth understanding of men’s victimization from physical aggression, not only highlighting the nature of violence itself but the fear it instills (E. A. Bates, 2020). This study also identified significant levels of verbal (e.g., yelling, screaming, shouting) and sexual aggression (e.g., sexual assault, orgasm control, being forced to penetrate their partner); findings supported by the work of Hines and Douglas (2016b) and Weare (2018) highlighted the existence and psychological impact on men who experience sexual aggression, including being “forced to penetrate” their partners. Work exploring violence toward GBT men both supports the findings above and highlights unique forms of violence within same-sex relationships, for example, the use of HIV status and “outing” to control victims, and the deliberate misuse of pronouns (Barnes & Donovan, 2018).

Importantly, using a qualitative online survey with men in opposite-sex relationships, E. A. Bates’ (2020) work also evidenced the use of coercive control, which hitherto had principally been investigated in female victims (Follingstad et al., 1990). Results demonstrated a variety of controlling tactics including control over personal freedom (e.g., limiting the use of mobile phones, engagement in social activities), manipulation and isolation (e.g., using children against a partner, threatening false allegations, falsifying pregnancy), gaslighting (e.g., making men doubt their perception of the world and sanity), humiliation (e.g., belittlement, lowering self-esteem), and promotion of fear and uncertainty (e.g., behaving unpredictably; E. A. Bates, 2020). Specific findings, for example, the use of legal and administrative aggression (e.g., the manipulation of legal systems against a partner), supported previous research demonstrating unique vulnerabilities for male victims (Hines et al., 2015; Tilbrook et al., 2010). Taken together, such results demonstrate the severity of abuse directed toward men, and the necessity for increased support and visibility for male victims.

As understanding of men’s experiences has increased, so has research on the impact and outcomes of abuse toward men. Indeed, while research has typically focused on outcomes for female victims (Straight et al., 2003) or sampling had not supported conclusions relating to male victims (Trevillion et al., 2012), studies have begun to demonstrate that DVA has demonstrable and long-term impacts on the physical and mental health of *both* men and women (Alejo, 2014; Coker et al., 2000, 2002). Indeed, Tsui (2014) found that, alongside physical injuries, men reported loss of self-worth and suicide ideation, while other studies have highlighted links with binge drinking (Hines & Straus, 2007), posttraumatic stress disorder (PTSD; Hines & Douglas, 2011), and overall poor health (Hines & Douglas, 2015, 2016a). Qualitative research again supports these findings, as men report significant and long-lasting physical and mental health outcomes including life-changing injuries (e.g., loss of vision, disability), PTSD, and feelings of extreme isolation and loneliness (E. A. Bates, 2019b). Findings relating to anxiety, and substance use and misuse, are also mirrored in work with GBT men (Bacchus et al., 2017). Moreover, men reported a lasting impact of abuse on the successful formulation of future relationships, as they felt unable to trust future partners, or were overly fearful. Importantly, for men who were also fathers, many reported that the relationship with their child(ren) was affected, for example, through experiences of alienation, parental relationship disruption, and the legal aggression described above (E. A. Bates, 2019b). Moreover, this use of systems, particularly family courts, had a substantial impact on the mental health of male victims (Berger et al., 2016).

The use of children, and the manipulation of father–child relationships, is also highlighted in work exploring aggression occurring toward men post separation. For example, Hines et al. (2007) describe men’s accounts of their partners threatening to remove children and/or manipulating systems against them after relationships had ended. Indeed, withholding contact from children, manipulation of parental relationship, and even using the child themselves as a vehicle for abuse (e.g., by encouraging negative behavior from the child to the father) are behaviors highlighted in qualitative work on this topic (E. A. Bates, 2019a). Other examples included continuing verbal aggression, escalation of previous abusive behaviors, and coercive control, including harassment and the use of false allegations (E. A. Bates, 2019a). Most crucially, this work further highlighted the lasting impact of abuse on men’s physical and mental health, and formulation of relationships.

Throughout much of the work exploring men’s experiences, the influence of damaging societal expectations and stereotypes surrounding masculinity is substantial. For example, in interviews with male victims, the ideas and expectations of men to be strong, stoic, dominant, in control of their emotions, and able to cope on their own (Connell, 2005) had a significant impact on how men viewed themselves as victims, or whether they even recognized their victimization at all (E. A. Bates, 2019b). Men perceived themselves as “weak” for “becoming a victim” and described how they felt society struggled to recognize them as victims (as men are typically characterized or seen as abusers). Indeed, others have suggested that broader gender stereotypes, combined with specific scripts regarding the nature of DVA, coalesce to minimize the experiences, visibility, and subsequent support available for male victims (Hine, 2019). Hine (2019) also highlights that the layers of stereotyping and prejudice are even more complex for GBT men, who additionally have to battle cultural heterosexism and homophobic attitudes (Herek, 1995). Such stereotypes, and the resulting minimization of male victims, may help to explain why female-on-male perpetrated abuse is viewed as less serious than male-on-female perpetrated violence, or same-sex DVA, and is less likely to have police intervention recommended (Ahmed et al., 2013; Feather, 1996; Hine, 2019; Poorman et al., 2003; E. P. Seelau et al., 2003; S. M. Seelau & Seelau, 2005; Sorenson & Taylor, 2005). It may also explain why the status of “victim” does not appear to carry the same credibility for men as for women (E. P. Seelau et al., 2003).

These detrimental stereotypes are also reflected in men’s experiences of help-seeking (Huntley et al., 2019) and attempting to leave relationships, as reactions from friends often reflect these negative attitudes (E. A. Bates, 2019b). Such findings reflect issues that are both synonymous with, and additional to, barriers to help-seeking identified for female victims (Fugate et al., 2005). Men have reported not being believed, being ridiculed, and have described how services were mocking of their experiences, or suggesting they were somehow responsible for the abuse (E. A. Bates, 2019b). Such results are complemented by findings that men tend to be blamed for their victimization to a greater extent than women by the general population (C. A. Taylor & Sorenson, 2005). Indeed, studies show that men often do not report their experiences of abuse because they themselves minimize it, and fail to label such experiences as domestic violence (Machado et al., 2016). They also report fear that they will not be taken seriously by authorities (Drijber et al., 2013). This is supported by previous work exploring men’s experiences of reporting DVA to the police, with men describing being recast as the perpetrator (in line with the “domestic violence stereotype”; Dutton & White, 2013), and that reporting their victimization resulted in a questioning of their masculinity (McCarrick et al., 2016). Responses from DVA support services have been shown to be equally problematic, with previous examinations concluding that the quality of service provision for male victims is, at best, mixed (E. A. Bates, 2019b; Huntley et al., 2019). Such results are potentially explained by previous assertions that the sector is a “female domain” which does not recognize men, and that the stigma associated with male victimization remains (Hester et al., 2012). This may go some way to explaining why many men feel they have to just “put up with” their abuse (E. A. Bates, 2019b), and that there is not equitable provision of, or access to, services and support (including safe housing/refuge spaces; Mankind Initiative, 2020). Men have also stressed that they feel inclined to stay with abusive partners to remain with and protect their children (E. A. Bates, 2020), as, again, there is little provision available which allows them to remove both themselves and their children away from abusive settings (Mankind Initiative, 2020). Studies also demonstrate that stigma is experienced by men reporting same-sex DVA (Calton et al., 2016; Laskey et al., 2019), which is represented in their negative experiences of help-seeking (Donovan & Barnes, 2019), including reporting to law enforcement (Finneran & Stephenson, 2013), and the difficulty they face in accessing services (Stiles-Shields & Carroll, 2015). Taken together, such studies explain why men report that being recognized as victims is of the utmost importance to them in their help-seeking journeys, and is the foundation on which all other help, support, and access can be achieved (Wallace et al., 2019a).

Consequently, attempts have been made to understand the current challenges in the delivery of DVA support for men, with a study conducted in Wales providing valuable insight. Researchers interviewed 20 managers and practitioners of DVA services supporting men, to explore what providers understand the needs of men who experience DVA to be (Wallace et al., 2019b). Themes developed highlighted that abused men face a battle against a “tide of recognition.” This lack of recognition hindered men’s ability to accept and recognize their experiences of abuse, which meant limited knowledge of provision and low numbers of men accessing support. Interestingly, these themes are reflective of the observations outlined above regarding the existence of stereotypes which limit the visibility of men, and in turn, limits the ability of services to operate effectively. This was then identified as part of the reason as to why resourcing for male services was low, alongside a broader lack of societal and governmental recognition for male victims (Wallace et al., 2019b). These findings are not dissimilar to those highlighted by providers supporting female victims. For example, funding is identified as a sector-wide issue, as all services struggle with underresourcing (Ishkanian, 2014). Moreover, L. Bates et al. (2001) found that there was a need to ensure women felt safe and understood in a supportive environment, and Tower (2006) found that among their group of social workers, family practitioners, and obstetrician–gynecologists, in-service education and the presence of institutional support decreased barriers and increased the use of screening. However, it is clear from the work of Wallace et al. (2019b) described above that, while female victims evidently face barriers to support, it is clear that men may face additional gender-specific barriers associated with masculine stereotypes and lack of available services.

However, despite important preliminary evidence from Wallace et al. (2019b), further research exploring the experiences of services working with male victims is still much needed. Such needs are further highlighted when acknowledging the small sample utilized in the Wallace et al. (2019b) study. Moreover, services in this study supported small numbers of men and thus may not capture the true range and scope of challenges faced by providers. It can also be argued that it is important to explore different types of service provision, again to fully determine the challenges faced by the sector, and to provide opportunities to further explore the experiences and outcomes of male victims in a larger, more diverse sample. Moreover, the psychological and emotional experiences and needs of service providers have yet to be explored in this context: a need highlighted by the literature highlighting the prevalence of vicarious trauma (including PTSD-like symptoms), and psychological burnout conducted on populations supporting female victims of DVA (Iliffe & Steed, 2000; Kulkarni et al., 2013). It is thus argued that research which continues to qualitatively explore the experiences of practitioners working with abused men will not only aide our understanding of the most efficient and effective ways of supporting male victims but also how best to support those providing services.

The present study utilized a qualitative interview design with call handlers at a U.K. domestic abuse helpline for men, described as providing a “confidential helpline . . . available for male victims of domestic abuse and domestic violence across the UK who are experiencing this abuse from their current or former wife or partner (including same-sex partner).” This organization was chosen for two principal reasons. First, the charity processes a substantial volume and variety of calls. Specifically, they answer approximately 1,400 calls per year, from male victims and those concerned about them (i.e., family and friends: 27%). They also receive over 200 calls a year from the police, councils, other support services, and those in the legal profession. Second, the charity provides services both directly to victims and to other specialist organizations, and is therefore uniquely positioned within the sector. Taken together, this presents an opportunity for truly unique insight into the challenges of supporting male victims of DVA. As outlined above, one of the principal challenges within this area of research is the overly narrow focus of previous research, specifically, on female victims of domestic abuse within opposite-sex relationships. With respect to diversity, as argued in this article, it is only right that the experiences of all individuals, regardless of sex and/or gender, or sexuality, are explored, so that services are best informed as to how to support these individuals. Therefore, a major strength of the research is that it seeks to explore the experiences of a largely “hidden” victim group, and to broaden the knowledge base in a way that is sensitive to the diversity of abuse victims.

This study had two principal aims: first, to provide more detailed information regarding the nature and context of abuse toward, and help-seeking experiences of, male victims, and second, to explore the practitioners’ experiences of supporting abused men. Importantly, all four call handlers at the charity were interviewed, representing a small-scale yet crucially important study for this sector and research area. In doing so, it was hoped that insight would be gained on men’s experience of DVA and the subsequent impact on themselves and those around them; barriers to disclosure and engagement with services for men, and how services seek to mitigate such obstacles; and the challenges of providing effective services for male victims, both within the context of running a helpline and the sector more broadly.

## Method

### Participants

A U.K.-based organization was approached, and all four call handlers provided interviews for this study. All were female, were aged between 43 and 69 years, and had worked at the organization for between 21 months and 14 years. Some had been involved in the sector (domestic violence support) for longer than their time at the current organization. Alongside providing frontline support, some occupied additional roles within the organization, including Manager and Independent Domestic Violence Advisor (IDVA). See Table 1 for an overview of demographic information, and participant’s chosen pseudonyms.

**Table 1.** Participant Demographic Information.

Pseudonym	Sex	Age (Years)	Role Within Organization (Alongside Call Handler)	Hours Worked (Per Week)	Time at Organization	Time in Sector Prior Domestic Violence Support	Qualifications/Training
Abigail	Female	58	Service Manager	40	14 years	N/A	IDVA, Safeguarding^a^, Supporting Male Victims^b^
Angela	Female	52	None	26	2 years 6 months	N/A	Safeguarding, Supporting Male Victims
Amy	Female	43	Head of Training	26	21 months	N/A	IDVA, DVSM, Safeguarding, Supporting Male Victims
Trish	Female	69	None	19.5	10 years	20 years (set up first male victim refuge in England)	Safeguarding, Supporting Male Victims

*Note.* IDVA = Independent Domestic Violence Advisor; DVSM = Domestic Violence Service Management.

a External Training Package. ^b^Internal Training Package.

### Materials and Procedure

A semi-structured interview guide contained an initial introduction designed to remind participants of their rights (e.g., to withdraw at any time) and to introduce participants to the format of the interview. This was followed by an opportunity for participants to share any information they wished about themselves personally, to encourage initial rapport building. A question allowing for free recall of their experiences followed, prompting participants to cover all/any aspects of their work they felt comfortable sharing. Subsequent questions and prompts were designed to probe four principal areas: (a) the characteristics and experiences of callers, (b) stereotypes and societal beliefs surrounding male victimization, (c) barriers to help-seeking and support, and (d) the experiences of call handlers. Participants’ interviews lasted between 45 and 90 min. Participants were offered breaks if required, and, once interviews were concluded, they were given a verbal and written debrief. Ethical approval for this study was granted by the university ethics panels at the first and second author institutions.

### Data Analysis

Interview recordings were transcribed verbatim by postgraduate research assistants and analyzed by the second and third authors using thematic analysis (TA). TA is a method used to identify, analyze, and report patterns/themes within a data set, a six-phase guide (Braun & Clarke, 2006) informed the basis of analysis and supported consistency. However, the guide was not rigidly used; a flexible approach was adopted to fit the research question and data (Patton, 1990). TA is concerned with understanding at group level or a descriptive portrayal and acquiring commonality among participant experiences. Themes characterizing men’s experiences of DVA have been developed in previous studies (Ansara & Hindin, 2010; E. A. Bates, 2019b, 2020; Hine, 2019; Wallace et al., 2019a). Identifying common threads across accounts given by the participants in this study offers insight of the phenomenon from the shared beliefs and attitudes of various populations. This lends itself to a framework typology of TA (Srivastava & Thomson, 2009), where a priori notions of the phenomena in question may be considered throughout the analytical process.

Analysis was not aided by software. Instead, the second and third authors independently coded interview transcripts by hand followed by several discussions about the developing codes to agree extracts, developing themes, and the thematic map. All notes, transcripts, and themes from analysis were maintained throughout ensuring reliability and providing an audit trail from raw data to interpreted results (Shaw, 2010).

## Results

**Figure 1.**
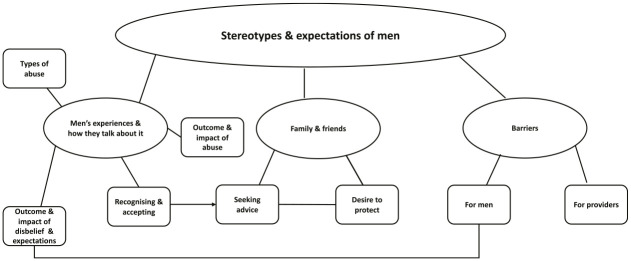
Study’s Thematic Map.

From our TA, we chose one superordinate theme, *Stereotypes and expectations of men*, and three overarching themes (with subthemes shown in brackets), *Men’s experiences and how they talk about it* (Types of abuse, Recognizing and accepting, Outcome and impact of disbelief and expectations, Outcome and impact of abuse), *Family and friends* (Seeking advice, Desire to protect), and *Barriers and challenges* (For men, For service providers). Subthemes are not necessarily independent and will have similarities or common ground between and within each of the three overarching themes. Figure 1 represents the final thematic map, depicting the themes and connections discussed in this section.

### Superordinate Theme: Stereotypes and Expectations of Men

The central superordinate theme, *Stereotypes and expectations of men*, influences all subsequent themes, in that the societal and internalized masculine stereotypes and expectations of men fed into all areas drawn from the analysis. Broadly, this theme represents unhelpful notions of gender and associated expectations of how men should behave, and indeed expect themselves to behave, and its negative effects on almost every aspect of their abusive experience (e.g., how they talk about it, types of abuse, its impact, and help-seeking and response):

I have guys that call me and say “I can’t be a victim of domestic abuse. How would I be a victim I am a police officer, I’m a judge, I’m a solicitor, I work in the field, I can’t be a victim.” Even though they know what is going on it’s like “I can’t talk to anyone, I can’t talk to anyone local” they say “I can’t engage with the service.” (T1 Abigail)

A man should provide a home, should provide a roof over the head of the family, they should provide a certain quality of life, good holidays, decent car or two [. . .]. They should pay mobile phone contracts for the children, and they should be the main breadwinner. They should be tough, as well as empathetic and all of those things. So I think where we at the moment we want a man to have an understanding we want men to be soft and gentle but we also still want that alpha male that sort of that provides and protects. So, we don’t perceive him to be a victim or able to be a victim. Whereas when we look at what a victim looks like, he doesn’t have a home, he’s weak, he’s not well dressed, he’s got poor mental health. So, it’s all the weak things that come along with that, that actually we see as not masculine within society. (T3 Amy)

The quotes above are representative of ingrained gender expectations which position men as providers, and that they are capable and strong. Interestingly, these notions appear entangled with changing expectations that men should, in modern society, also be “empathetic” and “gentle.” However, Amy acknowledges that, despite potential “softening” of the male gender role in the minds of participants, being a victim is still perceived as a weakness and that men are not readily accepted as victims. Abigail highlights the reluctance or inability of men to recognize and accept their victimization, which she perceives is influenced by a fear of losing or compromising their male/masculine identity.

This superordinate theme feeds into three overarching themes: *Men’s experiences and how they talk about it, Family and friends*, and *Barriers and challenges.*

#### Overarching Theme 1: Men’s experiences and how they talk about it

*Men’s experiences and how they talk about it* is supported by four subthemes: *Types of abuse, Recognizing and accepting, Outcome and impact of disbelief and expectations*, and *Outcome and impact of abuse.*

##### Types of abuse

Men calling the helpline experience a range of abuse, including physical, psychological, financial, sexual, and coercive control. Moreover, despite the severity of physical violence experienced, Abigail highlights that men are still reluctant to seek help. The behavior of men in “making excuses” for their perpetrators links to the following subthemes: *Recognizing and accepting* and *Outcome and impact of abuse*.

In the types of calls I would say the full gamut from people who are having small tiffs to people who have been stabbed and hidden that and bandaged it themselves and not gone to A&E or GPs and are still making excuses for their partners. (T1 Abigail)

I had one young man, he was not only the controlling behaviour and this was before coercive control was made a law, not that he would have recognised it anyway. He worked full-time he worked in a factory -type job and he would be given his pay check. She [partner] would come into his account and she did all the financials, she would go in take his pay check out of his account and put into the joint account to pay the bills [. . .]. She gave him £10 a week for his allowance to put fuel in the car. If he had to work any overtime and there wasn’t enough fuel to go in the car to go to and from work, he had to walk and this was 6 miles each way. That overtime money didn’t stay in his account it was then removed every month, that sheer control was there and there was no flexibility. (T1 Abigail)

Being Humiliated, belittling, being told they are nothing, they are not a man, they don’t earn enough, they don’t help around the house enough. They don’t, anything is not good enough to be quite honest. Financial abuse seems to be quite high as well, the fact that they seem to pay for everything the wife has got access to their money, but they haven’t access to the wife’s or partners money, they don’t know how much money they have actually money they have got. (T2 Angela)

Physical incidents they tend to be with weapons a lot of the time, so things being thrown at them, they’ve been hit with things, TV remotes, mobile phones, damage to property is a big thing. So people have stamped on their mobile phones or smashed their glasses or damaged stuff the laptops that type of thing. False accusations around having affairs that type of stuff is quite a big one. So, jealousy “where have you been?,” “what are you doing?,” monitoring and that type of thing. Harassing them at work. (T3 Amy)

From my experience of working front line with men its [sexual abuse] particularly within this service it’s more common than I ever imagined. (T3 Amy)

They [perpetrator] are very sorry afterwards that this [violence] has happened. Once it’s happened once it can happen again very easily and its easier the second time round, you know, and that’s how it goes on until it gets to a crescendo where they actually can’t cope or they are so badly injured it’s been taken out of their hands altogether the police have taken it out of their hands all together. (T4 Trish)

In their quote, Trish also highlights the cycle of the abuse: that physical abuse followed by apologies continues cyclically until the violence reaches a “crescendo” and men can no longer “cope.” This is particularly important in the context of male victims, as men who are unable to recognize abuse or seek support may remain in the abusive relationship for a longer period, as the “limit” to which abuse is tolerable may be higher.

Angela also refers to the use of children as a means of exerting power and control and a reason why men are unable to leave their abusive relationships:

Men obviously being, not leaving the relationship, because a lot of the times they are threatened that if they do leave, they will never see their children again [. . .] and that’s a big, it’s probably one of the, you know, the majority of the reasons why they won’t leave. The fact they say they will never see their children again, so and they are scared to leave because of that, and it a good way to keep them in the relationship. (T2, Angela)

##### Recognizing and accepting

This theme relates to men’s denial of their abuse. The issue of abused men being unable (or unwilling) to recognize and accept their victimization featured heavily in participant accounts. In part, this was accounted for by the lack of knowledge or awareness by men as to what constitutes DVA victimization, fear of not being believed, and shame of admitting being abused:

It’s being worried about the response, will they be believed? Will they be laughed at? A lot of it is to do with shame, you know, their man this shouldn’t be happening to a man and how will people take this. Once they have spoken to somebody like, you know, anybody like anybody somebody on the helpline, it gives them a bit more confidence [. . .] a lot of men that we get have been suffering abuse for years and haven’t told anybody, they’ve never reported it. (T4, Trish)

Mostly they’re phoning because they are the genuine victims and they want to experience validation to know that what they are experiencing is abuse. Because even if they’ve read it on the website they still want that voice on the other end of the phone to validate their experience. (T1, Abigail)

Yet, also highlighted was men not wanting their relationships to end and for their partners to receive support, which often kept them in their relationships and made them reluctant to take the significant step of labeling their experiences as abuse:

A lot of them [men] phone because they want help for their wife, they love them they don’t want the relationship or marriage to end. They want help for their wives. (T2, Angela)

##### Outcomes and impact of abuse

Participants referred to the extensive impact of abuse experienced by men. This included isolation, long-term physical problems, poor mental health (including feeling suicidal), and loss of contact with their children:

The isolation causes problems as well as they haven’t got anybody to talk to they haven’t got anybody to share with and for whatever reason they haven’t shared. I often say to people “it’s not that the wont report it’s that they can’t physically at that point they are not ready they are not ready to share.” (T4, Trish)

Lots of men talk about feeling low, quite a few talk about feeling suicidal some men have attempted suicide. Saying you know there’s been several attempts leading up to the telephone call and that type of thing [. . .] certainly mental health has had a massive impact [. . .]. Very often they will have either self-isolated or they will have been isolated from friends and family. Yeah, so there’s huge, huge effects on them as men and obviously, particularly if they’ve had children from a previous relationship as well. So the perpetrator will have stopped or alienated the children from a former relationship which really, really hurts or affects them. (T3, Amy)

Both Amy and Trish referred to men being isolated, the reasons for which are two-fold: first, isolation as a result of the abuse (e.g., being cut-off from family and friends), and second, isolation from a being unable to talk about their experience or admit/accept their victimization.

##### Outcome and impact of disbelief and expectations

This theme depicts the consequences of men not being readily accepted as victims of abuse by others (e.g., police and family courts). Disbelief that men can experience abuse, notions of what a victim is, a reluctance or inability to see themselves as victims/claim victim status, coupled with societal expectations of men may mean that men face further victimization when they seek help:

The police don’t see him as the victim, they see him as a perpetrator so it’s a continual cycle of the fact that they can’t be victims because they’re men so they must be perpetrators. I think society has always viewed men as being strong and women as being weak and I think some people assume that if you are man and you show any sign of weakness, then other men go “you must be gay.” So there’s that they don’t want to be cast as weak, they don’t want their masculinity to questioned so therefore its best not to say anything. (T2, Angela)

Amy refers to the bias of the “system” and how male victims are perceived in terms of both their victimization and expectations regarding contact with their children:

I think that men feel that there’s a bias, there in terms of the way the court and the system perceive them and It is perfectly ok for them not to have the [child] contact that they are rightly entitled to or not to have shared custody. I think as a society, you know, that fits with their experience, their experience is what they have seen happen to other people. (T3, Amy)

#### Overarching Theme 2: Family and friends

*Family and friends* is supported by two subthemes: *Seeking advice* and a *Desire to protect*. The theme represents the impact on families, their concerns and fears, and their attempts of help-seeking when they know their friend, son, or brother is being abused.

##### Seeking advice

Participants spoke of receiving frequent calls from family members, listening to their fears, attempts to help men recognize their abuse, and frustrations of not knowing how to help:

It was actually his daughter that convinced him [male victim] to ring us, he said “my daughters been telling me for years how terrible her mother is and she said that she was awful to her.” He said, “I didn’t want to believe that really what it was that I was experiencing was abusive behaviour.” (T1, Abigail)

I think on the helpline, the most frustrating thing for me is when its friends or family, when there asking “what can you do to save my son?” or “what can you do to help my brother”? We can give them guidance, but we can’t make a difference and think that for me that’s a massive challenge. Because obviously you want to help but actually it’s a case of waiting till that victim is ready to engage in [. . .] as much as we given them advise I think, I don’t feel that person necessarily has gone away with a positive result. (T3 Amy)

Abigail refers to an example whereby the daughter helped her father to recognize his abuse and to seek support, suggesting that family members may be instrumental in supporting men to recognize and seek help for their abuse. However, Amy remains frustrated by the limitations to the extent of support she can provide to families in the absence of the male victim being “ready to engage.”

##### Desire to protect

For participants, calls from concerned family seeking advice were the “most difficult.” Linking closely with the subtheme “Seeking advice,” staff face challenges in responding to family or friends if the victim themselves do not recognize or feel unable to admit their abuse. Underpinning these calls is the desire to protect those they love from experiences of abuse and seeking a solution or a means to help:

I would say that my most difficult callers are mothers and sisters because again we want to fix it we know what it is we recognise it and we want an agency to tell us how we make our loved one recognise what’s happening to them and recognise that it’s wrong. Their sons or brothers may not want to leave the relationship and they may not recognise that, they deep down know but may not want to admit what is happening is wrong. (T1, Abigail)

I didn’t hear from this mum for probably about 2 or 3 months and then when she did phone it was to tell me that he [male victim] killed himself. That is a body blow, you know, I never spoke to him, I never spoke him but you can imagine what his mum was feeling like can’t you. The effect it has on family and friends is huge, is absolutely huge and there’s nowhere to get help for themselves there’s nowhere for them to go for themselves. So we do the best that we can but there really isn’t a service out there that can help someone who is worried about somebody that’s being abused there really isn’t a service out there that deals with it. I don’t mind how often they have to phone me back I really don’t mind that [. . .]. They’ve just got to the point where they just need a bit of guidance how can I best help this but you can’t force anybody to do anything can you? (T4, Trish)

Trish recalls being informed by a mother of her son’s suicide, how this affected her, and her recognition of its impact on the victim’s family and friends. Trish also highlights a lack of specific support for family and friends of victims of abuse and reiterates feelings of frustration triggered by the lack of support, and feeling limited in the help they can provide without the victims acknowledging abuse.

#### Overarching Theme 3: Barriers and challenges

The overarching Theme 3, *Barriers and Challenges*, is supported by two subthemes: *For men* and *For service providers*. Participant accounts highlighted numerous barriers and challenges within the context of male domestic abuse: those specific to male victims and those specific to providing services for men.

##### For men

Men experiencing domestic abuse face numerous obstacles to seeking help and disclosing their abuse. Being able to recognize and accept their experience, acknowledge they need help, and fear of not being believed and of losing contact with their children are a few of the challenges raised in participant accounts. Below, Angela highlights knowing where to go, and financial implications are additional challenges experienced by men:

They [male victims] feel they can’t leave because they can’t afford to leave, and that’s a lot of the time why they stay in the relationship. They can’t afford to rent a house and pay the mortgage on the house they have got. Plus all the child maintenance, so that finically they feel trapped in the relationship. (T2, Angela)

Even the services that help men don’t always advertise that they help men so they are quite surprised when I say to them, if I signpost them to local service they go “oh I saw them but they only support women aren’t they?” and you explain “no they are not they support men as well.” But they [services] don’t make it clear that they support men as well. (T2, Angela)

When men do know where to go for support, they potentially face inconsistencies in the response they receive according to where they live:

It’s almost a postcode lottery to where you live in the county, cos different authorities and also individuals, but different authorities deal with things in a different way, you know. Sometimes it’s really aggressive so I mean its awful to say that just because your living in whatever area it is you know that’s you’re not getting the same the same response, and this is down to do with training. (T4, Trish)

##### For service providers

Providers of male victim services also face barriers and challenges. Abigail referred to several issues pertaining to the provision of support for men and increasing calls to the helpline:

I would say the challenges are the postcode lottery, so I mean you seen when we do our presentations that that right-hand side of the country that giant “C” there is a gaping big hole with no male refuges or safe houses. We know from our calls that people are and would use and if they were available. (T1, Abigail)

Things change, when we first started we get half a dozen calls a week now you get that in one morning and even that when you’re constantly being told that your helpline is engaged. (T1, Abigail)

Abigail further highlighted that increasing call volume is not solely related to increasing numbers of male victims calling. The helpline faces additional challenges of increasing inappropriate calls: calls from individuals needing mental health support who are unable to source specialist support and are instead contacting the helpline.

We are in the process of hiring another helpline person part time to try and determine where we need to overlap as we are at a point where we are getting a lot of calls that are not domestic abuse [. . .] We are picking up a lot of calls [that] should be going to mental health providers and services. Because mental health is underfunded and because they aren’t getting the support there and can’t go to the Health line they may need they go to any helpline possible. We are more high profile so the more high-profile you are the more calls you get. The more those people are when they should be according therefore their calls are a lot more challenging we are affirmation support but also a signposting service. (T1, Abigail)

Additional inappropriate calls add further pressure to an already busy helpline, the consequence of which requires hiring additional support.

Amy offered solutions to some of the challenges: more funding, tailored provision, separate support for men and women, training, and more proportionate funding and availability of support.

We need to resource more obviously, so more funding in to male services and to recognise that men don’t fit into women’s services and actually in some services the people that work there don’t comfortable support men in the same way as they support women. So actually, we do need to be looking into no one service fits all, it may be that we need a female and male service. Because men do respond differently to women, and so I don’t think sticking that pot of money together and saying “well all these IDVA’s and all these outreach workers and all these mental health workers can work with all victims.” Because actually, unless they are trained to work with all victims and to respond to victims on however they present then they are not going to get the best service. So you going to get someone who works really, really well with women, trying to support my man and failing and vice versa. So I think sometimes there is a need for separate services, the same level of funding and the same level of support and responding in different ways. (T3, Amy)

The participants also discussed challenges within service provision that related specifically to a helpline:

Well obviously listening to the stories and experiences, especially when you don’t know the outcomes, it can be quite, you know if you have had a few calls that day, it can be, you feel quite drained at the end of it. And then you can worry about callers, did they get out of that, and did they phone the service you suggest, did they, so you do worry about how they are. (T2, Angela)

The other challenge is you know sometimes is that you hear you hear really really quite difficult and quite horrific erm abuse that’s happening, erm and the hard part then is you never know what’s happened you don’t know the conclusion. (T4, Trish)

Both Angela and Trish discussed the emotional impact of supporting men: listening to their experiences of abuse, their worry about what has happened after the call, and their worry/frustration of not knowing the outcome. Trish highlights the contrast of this between hearing back from a caller and knowing that the support provided has made a difference:

If you do get that follow up phone call to say “thank you,” you know, and “this is what was done,” the support we gave has really helped. That doesn’t happen very often I must admit, but it’s really lovely when you actually get somebody that says “thank you, I’m now out of an awful relationship and you pointed me in the right direction” and I do get satisfaction out of that, I do. (T4, Trish)

## Discussion

The aims of this study were to provide more information on abused men’s experiences, and to explore practitioners’ experiences of working to support men on a domestic abuse helpline. This included exploring patterns in men’s experiences through the practitioners, understanding the nature of the barriers to disclosure and engagement with service for men, and finally, exploring the challenges of providing effective services for male victims within the sector. Uniquely, it is the first study to explore the views of those supporting a large and diverse sample of abused men, thus providing novel insights in this area of research. The overarching theme, and dominant narrative within the data, was around societal expectations of men, and the stereotypes that exist for male victims and for DVA more broadly. These expectations affected on all aspects of the abuse that men experienced, as well as on how they felt about help-seeking, and this was seen within the narratives of those who were supporting men and those asking vicariously for support.

In relation to the first aim, several key themes were found within this broader narrative. First, the participants described the significance and severity of the abuse that men experienced and were calling to talk about; this included severe physical violence, coercive and controlling behavior, financial, and sexual abuse. This also included describing cycles of abuse that have been seen within the previous literature on women’s experiences (e.g., Walker, 1979). Participants described how this abuse was causing these men significant mental and physical health problems, and some had also had their parental relationship manipulated. These findings support previous literature that discusses the range of DV that men experience within abusive relationships from both an international (e.g., Hines & Douglas, 2010; Hines et al., 2007) and U.K.-based (e.g., Barnes & Donovan, 2018; E. A. Bates, 2020; Wallace et al., 2019a) perspective, as well as the other studies that highlight the significant impact this has on them in terms of their health and well-being (e.g., see Bacchus et al., 2017; Coker et al., 2000, 2002; Hines & Douglas, 2016a). However, despite the range and seriousness of abuse, they described how many male callers struggled to recognize and accept their victimization; they felt shame and feared not being believed. This is again supported by previous research, as E. A. Bates (2019b) described how some men had been laughed at by friends and family when they had disclosed abuse, and had received further victimization from services where they had not been believed or in some cases had been blamed or accused of being perpetrators. Men have also been known to experience the impact of gender stereotypes within the Criminal Justice System (e.g., see Dutton & White, 2013; McCarrick et al., 2016), and indeed the mixed responses that they receive from these professionals are thought to contribute to what has been called a “bidirectional” lack of trust with police (Tsui, 2014). The participants in the current study described the isolation this left abused men experiencing: isolation from having had their social networks manipulated and reduced by their abusive partner, and also isolation at not feeling able to disclose.

The lack of recognition demonstrated by many men regarding their abuse may be due to the difficulties surrounding accepting a status as a “victim,” and how this is incompatible with other important stereotypes relating to both domestic abuse and masculinity. Indeed, it has previously been argued that conceptualizations of men within society as strong, self-reliant, and powerful means the message men are given about what it means to be a “man” is in contrast with asking for help or support (e.g., Hine, 2019; Vogel et al., 2011). This speaks to the existence of rigid gender role expectations placed upon men, commonly referred to as hegemonic masculinity (Connell, 2005; Connell & Messerschmidt, 2005), characterized by independence and stoicism. Moreover, the “domestic violence stereotype” (Dutton & White, 2013) positions men as perpetrators of unidirectional abuse toward women, thus not allowing a conceptualization of men as potential victims. In this sense, men face a “double jeopardy,” where they suffer prejudice through their victimization generally, and *as* men. Importantly, men’s difficulties around identifying and labeling abuse are likely to affect their help-seeking decisions (e.g., see Hine, 2019), and men report such detrimental stereotypes, for example, the challenge to their masculinity and their “invisibility” at services, as barriers to help-seeking (Huntley et al., 2019). This is reflected in studies which demonstrate that the general population also apply the status of “victim” to men and women unequally (E. P. Seelau et al., 2003), and that men may not be seen as the “ideal” victim, which may lead to more victim blaming attitudes (Meyer, 2016). Moreover, being labeled as “weak” and being seen as the abuser by society has been shown to significantly affect men in terms of *exacerbating* their abusive experiences (E. A. Bates, 2019b), and to create “secondary” and further victimization which intensifies trauma (Campbell & Raja, 2005). Indeed, service providers spoke about how traditional masculine stereotypes clashed with “new” societal expectations of men to display more traditionally feminine attributes, like empathy and emotionality (Franklin, 2012), thus providing even more confusion for men when processing their experiences. However, despite a reluctance or inability to identify as a victim, to access domestic abuse provision, men need to claim victim status. Once men access support, belief and validation helps men to accept their victimization (Wallace et al., 2019b).

Participants also described that they frequently spoke with men who were unable to seek help through fear of losing their children; indeed, this is thought to be the most common reason men who are fathers often stay within abusive relationship (e.g., see E. A. Bates, 2019a; J. C. Taylor et al., 2020). This is a fear often realized as abusive partners use legal and administrative aggression to manipulate services and family court systems to their advantage (e.g., Tilbrook et al., 2010). As such, further research on the use of parental alienation within the context of intimate partner violence is desperately needed. It is possible that one consequence of men’s reluctance to engage with services, through the fears listed above, is that those who are close friends and family are seeking advice on their behalf. While we know that the support of social networks such as these are key in both disclosure (e.g., Machado et al., 2016) and overcoming the impact (e.g., Safelives, 2019), there is no other literature to date that has found the extent to which these support networks are engaging in indirect help-seeking on behalf of victims. This is also not seen within either the women’s literature (nor is data available from women’s services for comparison) or indeed any other work that has examined men’s experiences; this vicarious support-seeking therefore remains unexplored. This is a novel and important finding, as while it may not be possible to overcome ingrained social stereotypes in the short term to enable men to disclose their experiences and seek help, it may be possible to work toward enabling and supporting these networks to facilitate disclosure and help-seeking by equipping them with the knowledge about how to do this. This is particularly important due to the participants’ accounts of the frustration these friends/family members felt at the men not recognizing their abuse, or the barriers they experience in being able to seek help themselves.

In relation to the second aim, this study provides further novel findings in exploring the experiences of service providers who work with male victims of DVA. Results showed that participants faced challenges in their practice which mirrored those identified for services working with women (e.g., the need to make clients feel safe, the need to educate on availability of services; L. Bates et al., 2001; Tower, 2006), alongside gender-specific challenges relating to the overcoming of damaging stereotypes relating to masculinity, abuse, and victimhood. They also reported struggling to cope with an increase in volume of calls without any increase in resource availability, and an increased volume of calls around men looking for mental health support that they were not qualified to provide. Importantly, while many female domestic violence services report issues with underresourcing (Ishkanian, 2014), the lack of funding provided for services supporting male victims is particularly acute, and is demonstrated by figures detailing government funding allocations; while the U.K. government do provide financial support to a helpline for male victims, fiscally it is not proportionate to the number of male victims seen within official statistics (e.g., one in three victims being male; Office for National Statistics, 2019). It is also important to note that, as services for male victims are awarded provision under the *Violence Against Women and Girls Strategy* in England and Wales, money is not always ring-fenced for services that solely support men. Indeed, some services in both England and Scotland currently run without any government funding and are reliant on donations or applying for alternative sources of fiscal stability. This supports Wallace et al.’s (2019b) observation that men face a battle against a societal “tide of recognition,” and their identification that this, coupled with a broader lack of societal and governmental recognition for male victims, could go some way to explaining why resourcing for male services is still so low.

The participants further discussed the impact of the work they did on them as service providers, referring to both the emotional impact and the specific challenges in delivering a helpline. In contrast to one-to-one or face-to-face support, participants providing telephone support felt a lack of follow-up leading them to worry about the well-being and outcome for those they had spoken with. We see from the wider literature that has explored service providers who work with women both in terms of DVA and sexual violence that vicarious trauma is a risk for those working with victims who experience trauma. Vicarious trauma (also known as compassion fatigue or secondary traumatic stress) is the negative physical and emotional reactions that can occur in response to indirect exposure to trauma with symptoms that often resemble PTSD type symptoms. Iliffe and Steed (2000) worked with counselors who supported both perpetrators and victims of DVA; they described feeling upset (physically and emotionally), and feeling drained by the accounts they had heard. This was something that was reported and had created issues over a longer period when they had repeated exposure. Moreover, of the 18 counselors who took part in this study, 12 felt burned out; indeed, psychological burnout is a known consequence of work stress (e.g., Kulkarni et al., 2013), but those in helping professions may experience this further due to feelings of guilt or poor boundaries (Lonne, 2003). Clearly, a priority for those providing services for those experiencing domestic abuse is to ensure that the physical and mental health of practitioners is protected, and that support is in place.

Taken together, the findings from the current study have important implications for those working with male victims of DVA, and the sector at large. Principally, results add further evidence not only on the severity of abuse experience by men but also the significant barriers and challenges they experience in help-seeking. These barriers exist on a number of levels, including personal barriers around masculinity and the male gender role, and systemically in how services are not available, or do not appear available, for men. As such, findings speak to a wider need around awareness-raising of male victims, both within the sector and society at large. Moreover, challenges within funding provision need to be addressed, so that, when male victims come to a point where they engage with services, more support is available. Crucially, there is a need for the promotion of services that are tailored for men. Funding cuts within the U.K. domestic abuse sector have left some service providers looking for cost-effective ways to delivery (Ishkanian, 2014), which will harm some victim groups being supported and also dilute knowledge and expertise within the area (Towers & Walby, 2012). Indeed, the availability and quality of service provision for male victims in the United Kingdom are mixed, and there is a need to ensure that they are delivered in a way that is appropriate for men (e.g., by ensuring anonymity and confidentiality; Huntley et al., 2019). These recommendations mirror those made by Hine (2019), which emphasize the breaking down of damaging stereotypes in various sectors to encourage men to “speak out” about their experiences, and for the provision of effective, gender-inclusive services, designed around the needs of male victims. In this sense, this study therefore seeks to diversify the current literature based on DVA by providing greater insight into the experiences of male victims, and to encourage the diversification of service provision to ensure that previously overlooked and disenfranchised victim groups (including GBT men) are catered for.

Despite the strengths of the current study, and the novel insight provided, there are some limitations. First, we explored the experiences of those working to provide a helpline for male victims of DVA within *one* organization in the United Kingdom, which means it is unlikely that the full breadth of experiences and challenges faced by service providers have been captured. Moreover, sampling from only one setting leaves testimony provided open to potential biases relating to the specific context, environment, and approach of that organization. However, this small-scale qualitative study has yielded an important insight into the experiences of service providers within this type of service provision, and the challenges that are faced for those who are supporting male victims of DVA only (as opposed to a service run for both men and women). Moreover, although this may appear as a small sample at face value, all four call handlers within this unique service were interviewed, providing a crucial specialist insight into challenges faced. This argument is supported by observations that qualitative research aims to capture rich sources of data, and that sample size is not an intrinsic requirement in producing worthwhile findings (Procter et al., 2010). In addition, as a helpline, this organization takes call from across the United Kingdom and is not therefore necessarily bound by geographical constraints. Nonetheless, future research to provide further support for these findings on a wider scale, and to explore where there may be different challenges or where other organizations may have found different ways of working, is needed. There is also further need for research to explore findings around indirect or vicarious support-seeking by friends and family members in more detail. Indeed, this finding is not something seen within the men’s literature before, nor is it mirrored within research with female victims. Therefore, research which allows for a better understanding of how third parties assist in help-seeking, particularly for men, may help service providers understand how to work with both male victims and their support networks to facilitate escape and recovery.

Principally, the results from this study demonstrate that stereotypes and expectations around men and masculinity pervade and influence not only men’s own perceptions of their abusive experiences but the way they seek help, and the decision to do so. Moreover, the same stereotypes affect the capacity of services to provide effective support to male victims, for example, by playing both a direct and an indirect role in the allocation of funding, and in minimizing awareness of services, and their necessity. It can therefore be argued that, before the sector as a whole can begin to effectively support men, in a consistent and robust way, such beliefs and prejudices, both within the sector and in society at large, must be challenged (Hine, 2019). As a short-term solution, findings above highlight the important role that third parties can play in enabling men to recognize and report their abuse. It is hoped that the results from this study in providing more information on the experiences of men themselves through the eyes of practitioners hopefully go some way to helping challenge some of the damaging attitudes around abused men (e.g., that men cannot be victims, or that they are not affected by abuse). However, there is a need to explore this further within the research to ensure all service provision (to men and their support networks) is representative of evidence-based practice.
